# Isatin Counteracts Diethylnitrosamine/2-Acetylaminofluorene-Induced Hepatocarcinogenesis in Male Wistar Rats by Upregulating Anti-Inflammatory, Antioxidant, and Detoxification Pathways

**DOI:** 10.3390/antiox11040699

**Published:** 2022-04-01

**Authors:** Nagwa G. Tawfik, Wafaa R. Mohamed, Hanan S. Mahmoud, Mohammed A. Alqarni, Ibrahim A. Naguib, Alzhraa M. Fahmy, Osama M. Ahmed

**Affiliations:** 1 Physiology Division, Zoology Department, Faculty of Science, Beni-Suef University, Beni-Suef 62521, Egypt; nagwatawfk2017@gmail.com; 2 Department of Pharmacology and Toxicology, Faculty of Pharmacy, Beni-Suef University, Beni-Suef 62514, Egypt; wafaa.mohamed@pharm.bsu.edu.eg; 3 Ecology Division, Zoology Department, Faculty of Science, Beni-Suef University, Beni-Suef 62521, Egypt; hanan.mohamed@science.bsu.edu.eg; 4 Department of Pharmaceutical Chemistry, College of Pharmacy, Taif University, P.O. Box 11099, Taif 21944, Saudi Arabia; m.aalqarni@tu.edu.sa; 5 Tropical Medicine and Infectious Diseases Department, Faculty of Medicine, Beni-Suef University, Beni-Suef 62521, Egypt; alzhraamohamed@med.bsu.edu.eg

**Keywords:** hepatocellular carcinoma, diethylnitrosamine, acetylaminofluorene, isatin, inflammation, apoptosis, oxidative stress

## Abstract

Hepatocellular carcinoma (HCC) represents around 85% of all known types of liver cancers and is estimated to be the fifth most common cause of cancer-related death worldwide. The current study assessed the preventive efficacy of isatin on diethylnitrosamine (DENA)/2-acetylaminofluorene (2-AAF)-induced hepatocarcinogenesis in male Wistar rats and investigated the underlying cellular and molecular mechanisms. HCC was initiated by intraperitoneal injection of DENA (150 mg/kg/week) for two weeks, followed by oral 2-AAF (20 mg/kg) every other day for three successive weeks. Oral isatin or vehicle (control) was administered at 25 mg/kg for 20 weeks during and following HCC induction. Isatin ameliorated the deleterious effects of DENA/2-AAF on liver function as evidenced by reduced serum levels of AST, ALT, total bilirubin, albumin, and liver tumor biomarkers (CA19.9 and AFP) compared to control DENA/2-AAF-treated rats. Histopathological evaluations demonstrated that isatin-mediated protection against hepatocarcinogenesis was accompanied by a decline in hepatic lipid peroxidation, a marker of oxidative stress, and enhanced antioxidant capacity, as evidenced by increased glutathione and superoxide dismutase expression. Isatin treatment also upregulated expression of the major stress-response transcription factor Nrf2 and the detoxifying enzymes NAD(P)H quinine oxidoreductase and glutathione-S-transferase alpha 2 and downregulated expression of the proliferation marker Ki67. Moreover, isatin significantly reduced the DENA/2-AAF-induced decrease in hepatic expression of anti-apoptotic Bcl2 and the DENA/2-AAF-induced increases in pro-inflammatory and pro-apoptotic factors (TNF-α, NF-κB p50, NF-κB p65, p53, and caspase 3). Thus, it can be concluded that isatin may protect against chemically induced hepatocarcinogenesis by enhancing cellular antioxidant, anti-inflammatory, and detoxification mechanisms, in part through upregulation of the Nrf2 signaling pathway.

## 1. Introduction

Hepatocellular carcinoma (HCC) is among the major causes of cancer-related mortality, accounting for 8.2% of all cancer deaths globally in 2018 [1]. Causative factors for HCC include infection by hepatotropic viruses such as HCV, HBV, and HAV, consumption of grains contaminated with mycotoxins, food- and water-borne nitrosamines, various air and water pollutants, nonalcoholic fatty liver disease, and alcoholic cirrhosis [2,3].

Diethylnitrosamine (DENA) is a potent hepatocarcinogen to both animals and humans [4,5] with many potential routes of exposure, including fried foods, tobacco smoke, alcoholic beverages, cosmetics, drug metabolism, and agricultural or industrial pollution [6,7]. The carcinogenicity of DENA is sufficient for use as an HCC inducer in rats when combined with 2-acetylaminofluorene (2-AAF) [8,9,10].

Current treatments for HCC include chemotherapy, surgical resection, and transplantation, but each has limitations [11]. Chemotherapy for HCC relies primarily on drugs that either inhibit DNA synthesis or modify its chemical structure. However, these agents damage both normal cells and tumor cells [12]. In addition, tumor resection and liver transplantation are not feasible in advanced stages of HCC [13,14]. An alternative strategy is chemoprevention [15,16], preferably using naturally occurring and/or synthetic agents with low inherent toxicity and low production costs [17].

Isatin (1H-indole-2,3 dione) is found in several medicinal plant species, including *Calanthe discolor*, *Isatis tinctoria,* and *Couroupita guianensis* [18,19]. Isatin and related analogs have a wide variety of demonstrated pharmacological activities, including anti-apoptotic [20], anticonvulsant [21], antiviral [22], cytotoxic [23], antimicrobial [24], anti-angiogenic [25], antiglycation [26,27], sedative [28], anxiogenic [29], antifungal [30], anti-tuberculosis [28], anti-HIV [24], antimalarial [31], and potential anticancer activities [32,33]. Furthermore, isatin is a potent antioxidant [34] and anti-inflammatory agent [35], and thus can both prevent free radical-induced carcinogenesis and kill cancer cells [24,32,36,37].

In the present study, we investigated the potential chemopreventive efficacy of isatin on DENA/2-AAF-induced hepatocarcinogenesis and the possible contributions of its antioxidant, anti-inflammatory, and chemical detoxification activities.

## 2. Materials and Methods

### 2.1. Drugs and Chemicals

Isatin, DENA, and 2-AAF were obtained from Sigma-Aldrich (St. Louis, MO, USA). Other used chemicals were of analytical grade.

### 2.2. Experimental Animals

Thirty adult male Wistar albino rats weighing 120–150 g were obtained from the Experimental Animal Facility, Faculty of Pharmacy, Nahda University, Beni-Suef, Egypt, and habituated to laboratory conditions for 7 days prior to experiments. Rats were housed under controlled temperature (25 °C ± 2 °C) and humidity (60% ± 5%) under a 12/12 h light/dark cycle with a standard diet and water ad libitum. The Animal Ethics Committee of the Faculty of Science, Beni-Suef University, Egypt, certified all procedures conducted on animals in this experiment (Approval Number: BSU/FS/2018/6).

### 2.3. Animal Grouping

**Figure 1:**
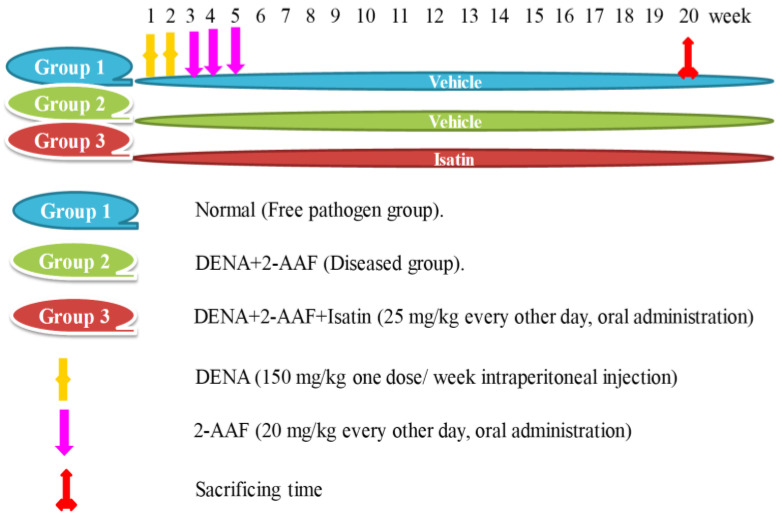
Illustration of the animal grouping and experimental design.

Rats were randomly allocated into three groups of ten (Figure 1). Rats of group 1 (Free pathogens group) were injected through the intraperitoneal route with an equivalent volume of isotonic saline (0.9% NaCl) and orally given 1% tween 80 and 1% CMC as vehicles. Rats within group 2 (DENA/2-AAF group) were intraperitoneally injected with DENA in saline (150 mg/kg) once a week for two weeks, then followed by oral administration of 2-AAF suspended in 1% tween 80 (20 mg/kg) for seven days after the last injection of DENA, every other day for 3 successive weeks [38]. Finally, group 3 (DENA/2-AAF + isatin) received DENA and 2-AAF as described in group 2 plus 25 mg/kg isatin in 1% CMC [39] by oral administration every other day from the beginning of the experiment to the end (20 weeks).

### 2.4. Blood and Liver Sampling and Analysis

At the end of the experiment, rats were sacrificed under anesthesia, and blood samples were withdrawn. Serum was separated from blood samples for analysis of liver function parameters and tumor biomarkers. The liver was excised and washed with cold, sterile saline. A piece (3 mm^3^) from each liver was fixed in 10% neutral buffered formalin for one day, then sectioned for histopathological analysis and immunohistochemical analysis of caspase 3 and p53. A second portion was homogenized in phosphate buffer at 25% w/v, then centrifuged at 3000 rpm for 15 min at −4 °C. The supernatants were retained and stored at −30 °C for subsequent analysis of hepatic malondialdehyde (MDA), reduced glutathione (GSH), and superoxide dismutase (SOD) activity. A third portion of the liver tissue was left at −30 °C for detection of nuclear factor erythroid 2-related factor 2 (Nrf2), NADP(H):quinone oxidoreductase (NQO1), glutathione-S-transferase alpha 2 (GSTA2), nuclear protein Ki67 (Ki67), B-cell lymphoma 2 (Bcl2), tumor necrosis factor-alpha (TNF-α), nuclear factor-kappaB p50 (NF-κB p50), and nuclear factor-kappaB p65 (NF-κB p65) by Western blotting.

### 2.5. Estimation of Serum Liver Function Tests Biomarkers

Aspartate transaminase (AST) and alanine transaminase (ALT) activities were determined in serum according to the methods of Bergmeyer et al. [40] and Gella et al. [41] using kits supplied by Biosystem S.A. (Costa Brava 30, Barcelona, Spain). Albumin and total bilirubin levels were estimated using reagent kits supplied by Biosystem S.A. (Costa Brava 30, Barcelona, Spain) based on Tietz [42] and Martinek [43], respectively.

### 2.6. Detection of Serum Levels of Tumor Markers

Carbohydrate antigen 19.9 (CA19.9) and alpha-fetoprotein (AFP) levels in serum were estimated by implementing ELISA kits provided by R&D systems (USA) according to the manufacturer’s instruction.

### 2.7. Detection of Liver Oxidative Stress Biomarkers

Malondialdehyde concentration in liver homogenate supernatant was measured by applying the protocol of Yagi [44], GSH content by the method of Beutler et al. [45], and SOD activity by the method of Marklund and Marklund [46].

### 2.8. Histopathological and Immunohistochemical Examination

Liver tissue samples fixed in 10% neutral buffered formalin for 24 h were dehydrated using ascending alcohol concentrations and then embedded in paraffin, followed by sectioning at 5 μm, staining with hematoxylin and eosin (H&E), and finally examined under light microscopy. Other fixed sections were processed for immunohistochemical staining of caspase 3 and p53. Furthermore, the sections were incubated with primary antibody in 3% H_2_O_2_, washed in phosphate buffer saline (PBS), incubated in peroxidase-conjugated secondary antibody for 30 min, washed again in PBS, and counterstained with hematoxylin. Immunostaining was evaluated by a qualified observer blinded to treatment history.

### 2.9. Western Blotting

For Western blot analysis, liver samples were homogenized in RIPA buffer and centrifuged to obtain clear supernatant. Bradford reagent was used to measure the total protein content. Then, 30 µg protein per gel lane was isolated by SDS-PAGE and moved to nitrocellulose membranes. Obtained membranes were blocked in Tris-buffered saline with Tween 20 (TBST) containing 5% non-fat milk powder, then incubated with primary antibodies against Nrf2 (Santa Cruz Biotechnology, Dallas, TX, USA), GSTA2 (Proteintech, Rosemont, IL, USA), NQO1 (Abcam, Cambridge, UK), Ki67 (EMD Millipore, Burlington, MA, USA), TNF-α (GeneTex, Irvine, CA, USA), Bcl2 (Santa Cruz Biotechnology, Inc., Dallas, TX, USA), NF-κB p50 (Santa Cruz Biotechnology, Inc., Dallas, TX, USA), NF-κB p65 (Santa Cruz Biotechnology, Inc., Dallas, TX, USA), and β-actin (Santa Cruz Biotechnology, Inc., Dallas, TX, USA). The β-actin housekeeping protein was used as a loading control to normalize the levels of protein detected by confirming that protein loading is the same across the gel. After washing the membranes with TBST, they were left to incubate with horseradish peroxidase-conjugated secondary antibodies (Novus Biologicals, Littleton, CO, USA) for one hour. Immunolabeling was detected using an enhanced chemiluminescence kit (BioRad, Hercules, CA, USA). Finally, obtained blots were scanned, and band intensities were quantified using ImageJ (NIH, Bethesda, MD, USA).

### 2.10. Statistical Analysis

GraphPad Prism 5 software was implemented for all statistical analyses. All results are expressed as mean ± standard error of the mean (SEM). The treatment group obtained means were compared by one-way analysis of variance (ANOVA) and then followed by Tukey–Kramer post-hoc tests for pair-wise comparisons. A *p* < 0.05 was considered statistically significant for all tests.

## 3. Results

### 3.1. Isatin Reduced DENA/2-AAF-Induced Increases in Biochemical Markers of Hepatic Dysfunction

Administration of DENA/2-AAF induced significant elevations in serum ALT and AST activities and total bilirubin levels while significantly reducing albumin levels compared to normal control rats. Concomitant treatment with isatin could significantly decrease serum ALT activity, AST activity, and total bilirubin levels while enhancing serum albumin levels compared to the DENA/2-AAF group (Table 1).

### 3.2. Isatin Reduced DENA/2-AAF-Induced Increases in Serum Tumor Marker Levels

**Figure 2:**
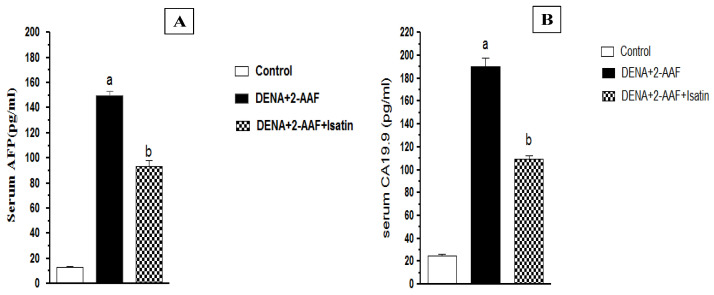
Isatin reduced serum tumor biomarker concentrations in DENA/2-AAF-treated rats. (**A**) Serum AFP concentration. (**B**) Serum CA19.9 concentration. Data are presented as mean ± SEM. ^a^ *p* < 0.05 vs. the control group. ^b^ *p* < 0.05 vs. the DENA/2-AAF-treated group.

Serum concentrations of the liver tumor biomarkers AFP and CA19.9 were significantly elevated in DENA/2-AAF-treated rats compared to control rats, while cotreatment with oral isatin significantly reduced serum AFP and CA19.9 compared to DENA/2-AAF treatment alone (Figure 2).

### 3.3. Isatin Suppressed Oxidative Stress and Enhanced Antioxidant Defense Capacity in HCC Model Rat Liver

**Figure 3:**
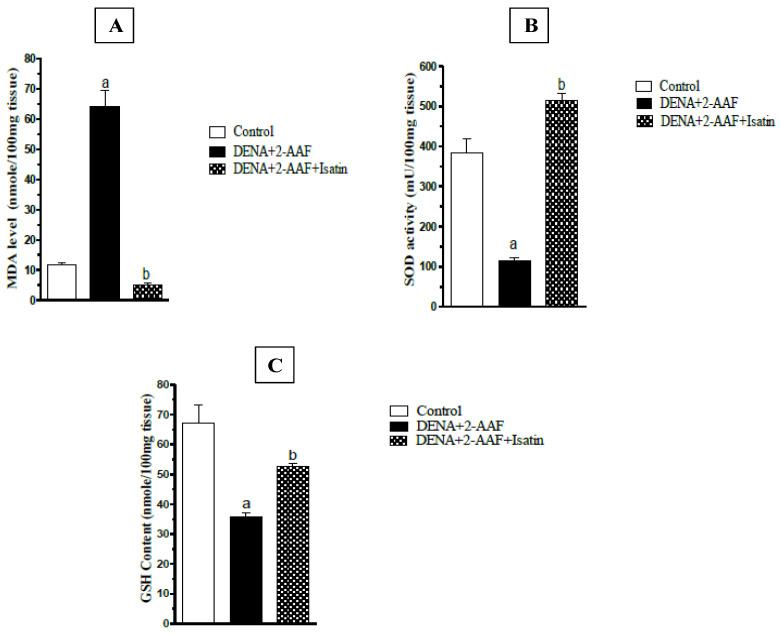
Isatin suppressed hepatic oxidative stress in HCC model rats. (**A**) Liver MDA concentration. (**B**) SOD activity. (**C**) GSH concentration. Data are presented as mean ± SEM. ^a^ *p* < 0.05 vs. control group. ^b^ *p* < 0.05 vs. DENA/2-AAF group.

Administration of DENA/2-AAF significantly elevated liver MDA content, a marker for membrane lipid peroxidation, and reduced liver GSH content and SOD activity compared to control rats. Consistent with reduced oxidative stress and enhanced antioxidant capacity, isatin cotreatment significantly reduced hepatic MDA content and could increase both GSH content and SOD activity compared to rats administered DENA/2-AAF alone (Figure 3).

### 3.4. Isatin Suppressed DENA/2-AAF-Induced Elevations of the Pro-Inflammatory Factors TNF-α, NF-κB p50, and NF-κB p65 in Rat Liver

**Figure 4:**
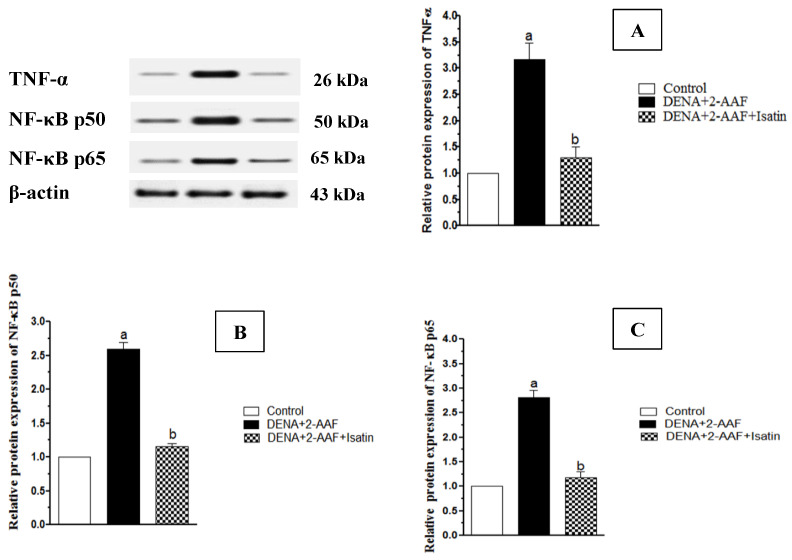
Isatin reversed DENA/2-AAF-induced upregulation of TNF-α (**A**), NF-κB p50 (**B**), and NF-κB p65 (**C**) protein expression in rat liver. Typical Western blots are shown (upper left panel). Data are presented as mean ± SEM. ^a^ *p* < 0.05 vs. control group. ^b^ *p* < 0.05 vs. DENA/2-AAF group.

Rats administered DENA/2-AAF exhibited significantly elevated liver expression levels of the pro-inflammatory factors TNF-α, NF-κB p50, and NF-κB p65 compared to control rats (*p* < 0.05). Consistent with anti-inflammatory activity, isatin cotreatment significantly reduced TNF-α, NF-κB p50, and NF-κBp 65 protein expression levels compared to DENA/2-AAF treatment alone (Figure 4).

### 3.5. Isatin Enhanced Expression of Detoxification Pathway Proteins Nrf2, NQO1, and GSTA2 in HCC Model Rat Liver

**Figure 5:**
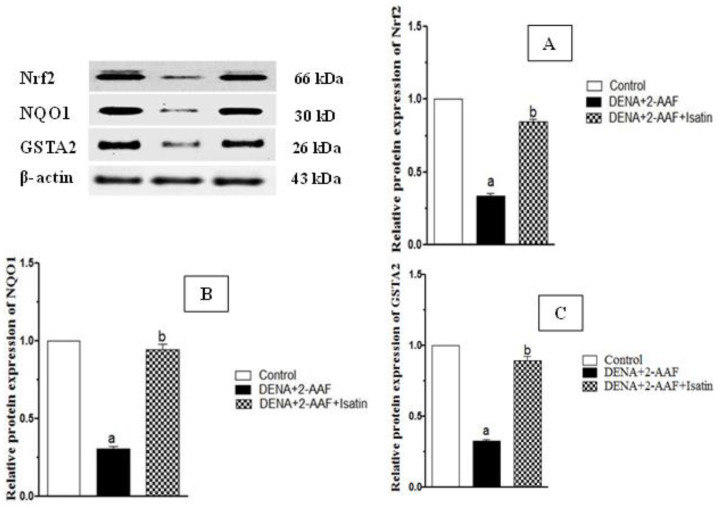
Isatin enhanced Nrf2 (**A**), NQO1 (**B**), and GSTA2 (**C**) protein expressions in DENA/2-AAF-treated rat liver. Typical Western blots are shown (upper left panel). Data are presented as mean ± SEM. ^a^ *p* < 0.05 vs. control group. ^b^ *p* < 0.05 vs. DENA/2-AAF group.

Hepatic expression levels of the detoxification pathway proteins Nrf2, NQO1, and GSTA2 were significantly reduced in DENA/2-AAF-treated rats compared to controls, while expression levels of all three proteins were upregulated by concomitant isatin treatment (Figure 5). In fact, isatin increased expression to near-control group levels.

### 3.6. Isatin Suppressed DENA/2-AAF-Induced Upregulation of Ki67 Protein Expression in Rat Liver

**Figure 6:**
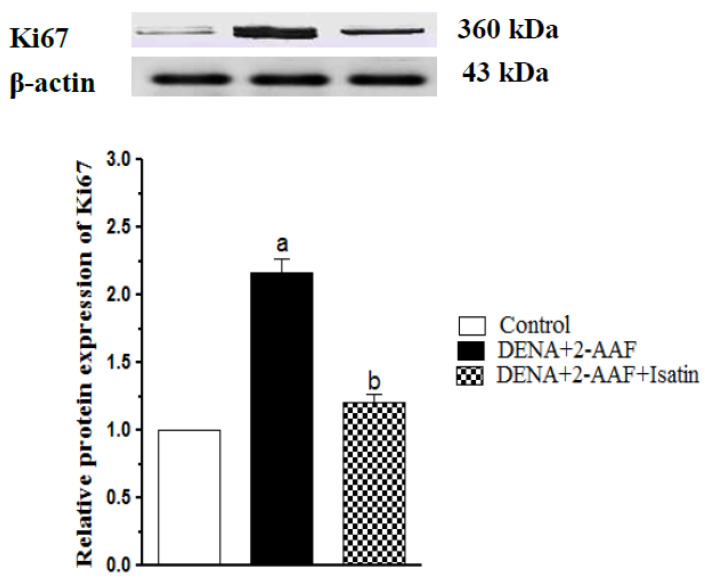
Isatin reduced Ki67 protein expression in DENA/2-AAF-treated rats. A typical Western blot is shown (upper panel). Data are presented as mean ± SEM. ^a^ *p* < 0.05 vs. control group. ^b^ *p* < 0.05 vs. DENA/2-AAF group.

The expression of Ki67 protein was significantly upregulated in the liver of DENA/2-AAF-treated rats (*p* < 0.05). Consistent with suppression of cell proliferation, isatin significantly downregulated hepatic Ki67 expression compared to DENA/2-AAF administration alone (Figure 6).

### 3.7. Isatin Reversed the DENA/2-AAF-Induced Reduction in Liver Bcl2 Expression

**Figure 7:**
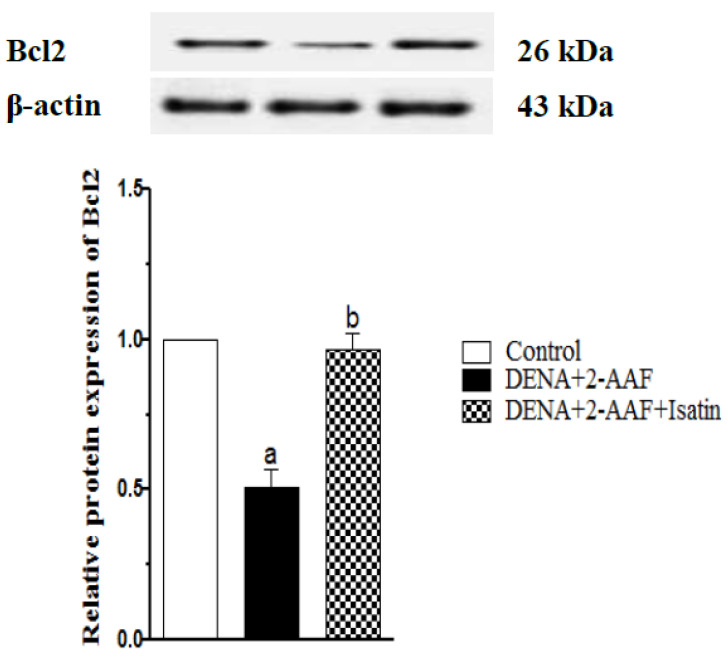
Isatin reversed the DENA/2-AAF-induced downregulation of hepatic Bcl2 protein expression. A typical Western blot is shown (upper panel). Data are presented as mean ± SEM. ^a^ *p* < 0.05 vs. control group. ^b^ *p* < 0.05 vs. DENA/2-AAF group.

Administration of DENA/2-AAF significantly reduced hepatic expression of the anti-apoptotic factor Bcl2 compared to control rats (*p* < 0.05), while isatin cotreatment reversed this effect (Figure 7).

### 3.8. Isatin Suppressed the DENA/2-AAF-Induced Upregulation of Pro-Apoptotic Factors p53 and Caspase 3 in Rat Liver

**Figure 8:**
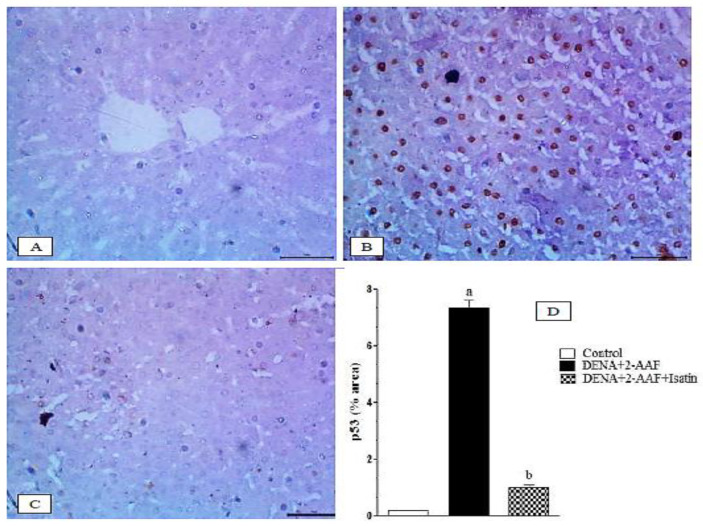
Isatin reduced the DENA/2-AAF-induced p53 upregulation in rat liver. (**A**) Immunohistochemically stained section depicted weak expression of p53 in normal rats. Livers of DENA/2-AAF-treated rats (**B**) showed upregulated p53 immunoexpression compared to normal control livers (**A**), while livers of rats cotreated with DENA/2-AAF and isatin (**C**) showed significantly reduced p53 overexpression compared to DENA/2-AAF treatment alone. Percent values of immunohistochemically stained areas (**D**) are presented as mean ± SEM. ^a^ *p* < 0.05 vs. control group. ^b^ *p* < 0.05 vs. DENA+2-AAF.

The nuclear and cytoplasmic p53 expression in the liver of normal rats (Figure 8A) exhibited a very weak expression of p53, while DENA/2-AAF treated rats showed a strong immunohistochemical reaction to nuclear p53 (Figure 8B). The cotreatment of DENA/2-AAF treated rats with isatin significantly suppressed the nuclear p53 expression (Figure 8A,D).

**Figure 9:**
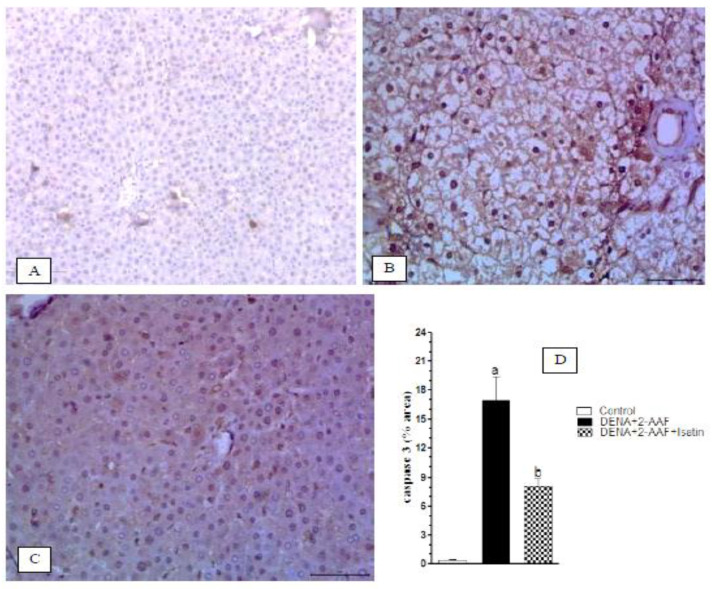
Isatin reduced the DENA/2-AAF-induced caspase 3 upregulation in rat liver. (**A**) Immunohistochemically stained section depicting weak expression of caspase 3 in normal rats. (**B**) Liver of DENA/2-AAF-treated rats showed upregulated caspase 3 immunoexpression compared to normal control livers (**A**), while the livers of rats cotreated with DENA/2-AAF and isatin (**C**) showed significantly reduced caspase 3 overexpression. Percent values of immunohistochemically stained areas (**D**) are presented as mean ± SEM. ^a^ *p* < 0.05 vs. control group. ^b^ *p* < 0.05 vs. DENA+2-AAF.

The cytoplasmic caspase 3 expressions in the liver of normal, DENA/2-AAF treated rats, and DENA/2-AAF treated rats supplemented with isatin are depicted in Figure 9A–C, respectively. The normal rats exhibited a very weak expression of liver caspase 3 (Figure 9A). Administration of DENA/2-AFF induced significant increases in expression levels of caspase 3 in the liver (Figure 9B,D), while cotreatment with isatin suppressed these effects (Figure 9C,D).

### 3.9. Isatin Attenuated DENA/2-AAF-Induced Histopathological Changes

**Figure 10:**
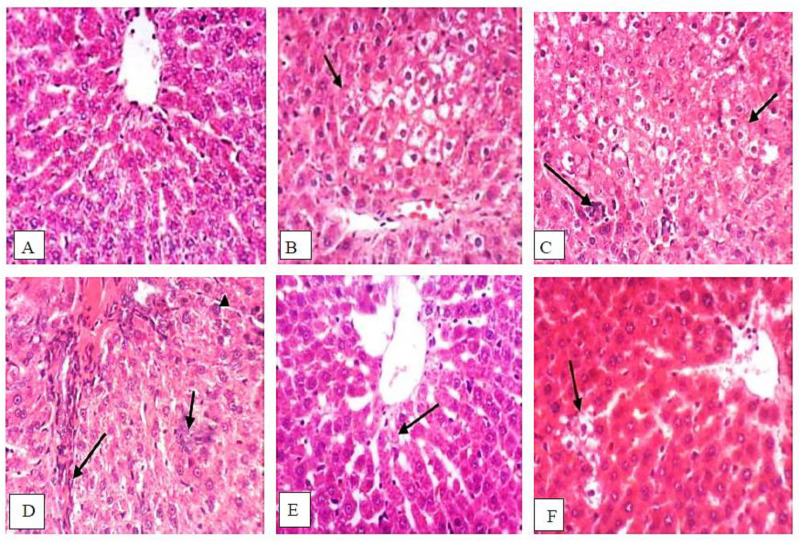
Isatin partially prevented the histopathologic changes induced by DENA/2-AAF. (**A**) Normal histological structure of the hepatic lobule in sections from control group rats. (**B**–**D**) Severe histopathological changes in liver sections from DENA/2-AAF-treated rats, including large clear hepatocellular foci surrounded by proliferating oval cells, fibroblast proliferation, and karyomegaly of hepatocytic nuclei. Moreover, HCC model liver also contained many cells with centrally located nuclei and several large hepatocellular foci with pale cytoplasm. (**E**,**F**) Isatin cotreatment greatly reduced but did not eliminate these histopathological signs (H&E; 400×).

Liver sections from normal control group rats exhibited typical histological architecture (Figure 10A), while liver sections from DENA/2-AAF-treated rats showed large, clear, hepatocellular foci surrounded by proliferating oval cells (Figure 10B,C) as well as fibroblast proliferation and karyomegaly of hepatocytic nuclei (Figure 10D). These histopathological signs of HCC were markedly reduced in rats cotreated with DENA/2-AAF and isatin; however, small focal abnormalities in hepatocellular architecture, slight karyomegaly of scattered hepatocyte nuclei, and sporadic necrotic cells were still detected (Figure 10E,F).

## 4. Discussion

Liver cancer is considered the second most lethal form of cancer [47,48], and global prevalence continues to rise [49,50]. Therapies for HCC, including radiation, chemotherapy, surgical resection, and ablation, can have undesirable side effects or minimal efficacy due to delayed diagnosis and induced drug resistance [33,51]. Therefore, new chemotherapeutic agents of natural sources are urgently needed to prevent or slow HCC progression. Isatin (1H-indole-2,3-dione) has garnered considerable interest due to its anticancer activities and is considered a novel candidate for low-toxicity tumor therapy [18,52,53]. In this study, we confirm in a rat model that isatin can reduce the deleterious biochemical alterations and histopathological signs of HCC, in part by upregulating defenses against oxidative stress, inflammation, and chemical toxicity.

Administration of DENA/2-AAF could induce liver injury, as illustrated by increased serum ALT, AST, and total bilirubin levels, and reduced serum albumin levels, in accord with previous studies [9,54,55,56,57,58,59,60]. Increased serum ALT and AST levels are due to their leakage from injured hepatocytes. Isatin treatment reduced serum ALT, AST, and total bilirubin levels, while significantly increasing serum albumin levels—effects that may be attributed to the preservation of hepatocyte membrane integrity. In support of this attribution, a decrease in hepatic MDA was found in rats receiving isatin, and previous studies have reported similar protective effects of isatin on liver function [61].

As expected, DENA/2-AAF treatment also increased serum levels of the tumor markers AFP and CA19.9, in accord with previous studies [4,62,63]. Tao et al. [64] also found increased CA19.9 in the serum of HCC patients. Isatin treatment of DENA/2-AAF-treated rats reduced both elevated AFP and CA19.9 levels, consistent with antitumor activity. Further, isatin reduced the expression of Ki67, a nuclear marker of proliferation frequently overexpressed in aggressive forms of cancer.

**Figure 11:**
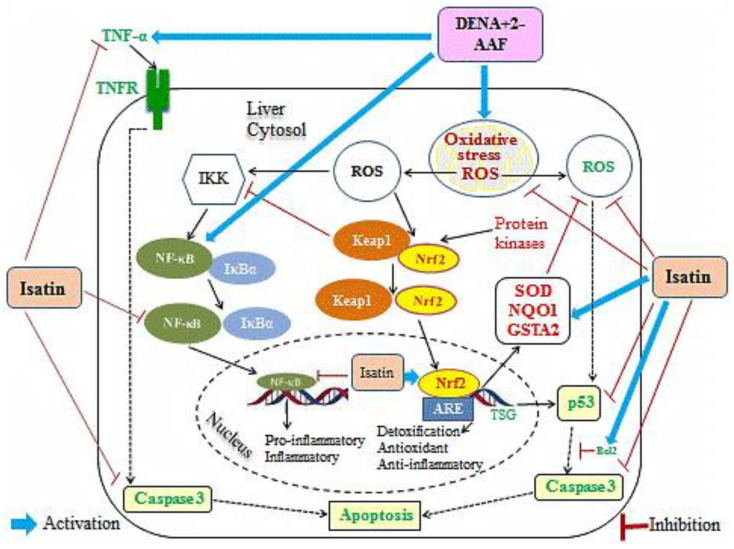
Schematic figure showing the mechanisms underlying isatin actions on oxidative stress, inflammation, and apoptosis in DENA/2-AAF-treated rat liver.

Histopathological examination of liver sections from DENA/2-AAF-treated rats revealed large clear hepatocellular foci surrounded by oval cell proliferation, fibroblast proliferation, and karyomegaly of hepatocytic nuclei, in conformity with previous reports [38,65]. Isatin markedly attenuated these signs of histopathology, likely due to the combined effects of pro-inflammatory factor suppression, enhanced expression of detoxification enzymes, and increased antioxidant capacity, as evidenced by the present study (Figure 11).

Malignant cells are generally more resistant to apoptosis, and numerous studies have emphasized the importance of altered apoptosis to tumor growth and development, although the pattern of alteration varies among different cancers, as demonstrated by Guo et al. [9,66,67,68]. Apoptosis is infrequent in normal hepatic tissues, while it was proven that HCC tissues show higher rates [66,69]. Therefore, the functions of apoptotic genes may differ in HCC, and indeed, the protective effects of isatin were associated with enhanced expression of the anti-apoptotic factor Bcl2 and reduced expression levels of the pro-apoptotic factors caspase 3 and p53. These findings are in accord with Mo’men et al. [70], who reported elevated p53 and caspase 3 after chronic DENA exposure, and also with de La Coste et al. [71] and Ahmed et al. [9], who discovered that Bcl2 expression prevents HCC by slowing the replication of transformed cells. In contrast to our findings, however, others have found that the inactivation of apoptosis by reducing p53 and caspase 3 facilitates DENA-induced hepatocarcinogenesis [72]. The reducing effect of isatin on caspase 3 was in concurrence with other publications [73], which revealed that the isatin-sulphonamide derivatives exhibited inhibitory effects on caspase 3 activities. As indicated in schematic Figure 11 and supported by our results, the DENA/2-AAF exposure stimulates the extrinsic apoptotic pathway by upregulating TNF-α, which activates TNFR, and stimulates the intrinsic pathway of apoptosis by increasing oxidative stress and p53 and caspase 3 expression and suppressing Bcl2 expression. On the other hand, the cotreatment with isatin reduced apoptosis by affecting both pathways (Figure 11).

DENA is a potent environmental carcinogen thought to induce transformation by generating reactive oxygen species (ROS) [74] that damage proteins, lipids, and (especially) DNA [75]. It is noteworthy that in many publications, oxidative stress and excessive generation of ROS play a crucial role in the initiation of hepatocarcinogenesis by causing DNA damage and mutations [9,76,77].

Indeed, the DENA/2-AAF administration induced a redox imbalance as evidenced by significant decreases in SOD activity, GSH content, and a significant increase in hepatic MDA accumulation. Consistent with our findings, Ahmed et al. [9], Mo’men et al. [70], and Yassin et al. [77] found that DENA induced oxidative stress, paving the way to hepatocellular carcinoma.

In turn, administration of isatin to DENA/2-AAF-treated rats, in the present study, significantly increased SOD activity as well as GSH content and significantly reduced hepatic MDA content. These findings are consistent with Premanathan et al. [34] and Bharathi Dileepan et al. [78], who demonstrated the potent antioxidant induction properties of isatin. The antioxidant activity, in general, was explained by the presence of the enolic hydroxyl group at the second position due to keto-enol tautomerism between NH and C=O [79]. The isatin also reduced the oxidative stress in the liver by reinforcing the antioxidant defense system through increases in the liver GSH content and activation of antioxidant enzymes, including SOD, GASTA1, and NQO1, as indicated by the results of the present study. The reduction in oxidative stress and ROS production by isatin has an important role in the mediation of anti-inflammatory and anti-apoptotic effects of isatin (Figure 11).

To study potential molecular mechanisms for the anti-carcinogenic activity of isatin, we focused on Nrf2, a transcription factor that binds to the antioxidant response elements (AREs) of multiple genes involved in protection against xenobiotics and pro-oxidant chemicals [80]. DENA upregulates NF-κB p50 and NF-κB p65 expressions while isatin reduces expressions, and DENA reduces Nrf2 expression while isatin counteracts these actions, suggesting cross-talk between Nrf2 and NF-κB [81] (Figure 11). Indeed, NF-κB was previously reported to act antagonistically on Nrf2 [82]. The increase in NF-κB expression induced by DENA/2-AAF is in accord with published studies [83,84]. Similarly, downregulation of Nrf2 by DENA administration has been reported in mice [81]. Downregulation of Nrf2 signaling is a frequent outcome of extreme and sustained reactive oxygen species production [67].

Administration of DENA/2-AAF also decreased NQO1 and GSTA2 expression, both of which are important for detoxification of xenobiotics and thereby reducing oxidative stress [85]. Administration of isatin significantly upregulated both NQO1 and GSTA2 in addition to Nrf2, which have contributed to the enhanced antioxidant capacity.

Nuclear antigen Ki67 expression is a biomarker for cancer staging [86], and its existence through all active phases of the cell cycle but not in resting cells (G0) presents it as an excellent biological marker for cellular proliferation and HCC prognosis [68,87].

Administration of DENA/2-AAF markedly enhanced Ki67 protein expression, consistent with previous findings by Pižem et al. [88], Raghunandhakumar et al. [89], and Yassin et al. [77], demonstrating that Ki67 expression is elevated in HCC, while expression was reduced by isatin, suggesting that the effects on antioxidant capacity, inflammation, and detoxification ultimately serve to reduce carcinogenesis [90,91].

The majority of cancerous tissues manifest signs of inflammation associated with specific inflammatory cytokines, remodeling, and angiogenesis [92]. Isatin treatment significantly decreased levels of the inflammatory markers NF-κBp50 and NF-κBp65, and NF-κB is considered an important link between HCC and inflammation. Indeed, suppression of NF-κB signaling can influence the efficacy of anticancer treatment. Treatment with DENA/2-AAF also increased TNF-α, a reliable marker of inflammation in hepatic carcinoma [93], while isatin cotreatment significantly reduced hepatic TNF-α, confirming an anti-inflammatory effect [94] associated with protection against hepatocarcinogenesis. These results are also in harmony with a previous study demonstrating reduced preneoplastic lesion and liver tumor formation in TNF-α receptor knock-out mice [95].

We conclude that isatin suppressed hepatocarcinogenesis by enhancing antioxidant defenses, suppressing oxidative stress, repressing inflammation, promoting resistance to apoptosis, and activating detoxification pathways (Figure 11). However, further studies are required to scrutinize all functional perspectives, including metabolic aspects of isatin in cancer and their interlinks in collaboration with researchers in the fields of genomics, proteomics and metabolomics, computational biology and bioinformatics, in silico studies, molecular docking and interactomics considering Gene Ontology (GO) and Kyoto Encyclopedia of Genes and Genomes (KEGG) Enrichment Analysis. Moreover, studies on other HCC models are warranted to provide the basis for clinical research on the therapeutic efficacy of isatin against HCC and other cancer types.

## Figures and Tables

**Table 1:** Effects of isatin on serum ALT, AST, total bilirubin, and albumin levels in DENA/2-AAF-administered rats.

Groups	ALT (U/L)	AST (U/L)	Total Bilirubin (mg/dL)	Albumin (g/dL)
**Control**	19.30 ± 1.44	71.50 ± 3.753	0.52 ± 0.03	3.98 ± 0.15
**DENA+2-AAF**	77.50 ± 3.81 ^a^	152.1 ± 7.17 ^a^	0.94 ± 0.07 ^a^	1.92 ± 0.19 ^a^
**DENA+2-AAF** **+isatin**	44.50 ± 3.35 ^b^	125.2 ± 2.03	0.45 ± 0.03 ^b^	3.10 ± 0.02 ^b^

Each value represents the mean ± SEM. ^a^ *p* < 0.05 vs. the control group. ^b^ *p* < 0.05 vs. the DENA+2-AAF group.

## Data Availability

Data is contained within the article.
